# Simultaneous Study of the Combined Effect of Gliding Arc Plasma and *Zataria multiflora* Essential Oil on Pathogens Inactivation and Quality Changes in Sliced Iranian White Cheese

**DOI:** 10.1002/fsn3.70320

**Published:** 2025-06-12

**Authors:** Mahdieh Raoofi Asl Soofiani, Negin Noori, Afshin Akhondzadeh Basti, Hassan Gandomi, Hamed Ahari, Mohammadreza Khani

**Affiliations:** ^1^ Department of Food Hygiene and Quality Control Faculty of Veterinary Medicine, University of Tehran Tehran Iran; ^2^ Department of Food Science and Technology, Science and Research Branch Islamic Azad University Tehran Iran; ^3^ Laser and Plasma Research Institute Shahid Beheshti University Tehran Iran

**Keywords:** antimicrobial, cold atmospheric plasma, essential oil, sensory properties, shelf life, white cheese

## Abstract

This study aimed to inactivate *Escherichia coli O*_*111*_, *Listeria monocytogenes* , and *Aspergillus flavus* on sliced Iranian white cheese by using the combination of gliding arc nonthermal plasma (GAP) and *Zataria multiflora* essential oil (ZEO). The cheese samples were exposed to different levels of GAP (0, 2, and 5 min) and ZEO (0 and 100 ppm) and stored at 4°C for 60 days. The extraction yield of ZEO based on dry weight was 1.66%. Carvacrol (33.85%) was the most important compound in ZEO. During the storage period, *E. coli* and *L. monocytogenes* counts increased in all samples. However, the most effective inhibition of *E. coli* and *L. monocytogenes* was observed in samples treated with 100 ppm ZEO combined with 2 min of GAP, and 100 ppm ZEO combined with 5 min of GAP, respectively, resulting in increases of only 1.35 and 1.8 log CFU/g over the storage period. In terms of the growth inhibition percentage of *A. flavus* , the lowest inhibition was detected in the presence of essential oil alone, while the highest inhibitory effect resulted from 5 min of plasma treatment combined with essential oil. GAP treatment also reduced the pH value of the cheese while increasing thiobarbituric acid reactive substances (TBARS). The control sample showed the lowest pH and the highest TBARS during storage. The highest overall acceptance sensory properties score was observed in cheese samples treated with 2 min of GAP and 100 ppm ZEO. According to the results of microbial, chemical, and sensory tests, Iranian white cheese samples treated with 2 min of GAP and 100 ppm ZEO can be used as a novel technique for sterilization and extending the shelf life of white cheese.

## Graphical abstract

This study aimed to inactivate two pathogens on Iranian white cheese by using the combination of gliding arc nonthermal plasma (GAP) and *Zataria multiflora* essential oil (ZEO). The cheese samples were exposed to different levels of GAP and ZEO, and samples were stored. According to the results, Iranian white cheese samples treated with 2 min of GAP and 100 ppm ZEO can be used as a novel technique for sterilization and extension of the shelf life of white cheese.

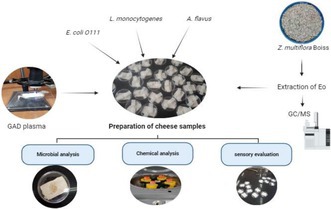

## 1 Introduction

Food safety is a major and common issue in the global food industry. Microbial spoilage of foodstuffs is a significant concern for academic researchers, food manufacturers, and consumers. Cheese is one of the most popular foods, and with its growing consumption, disease outbreaks related to cheese have increased worldwide. Many documented incidences of foodborne pathogens have been identified in cheeses. For instance, Makino et al. (2005) and Koch et al. (2010) reported that *L. monocytogenes* caused an outbreak of listeriosis in Japan associated with washed‐type cheese. Additionally, commercial cheeses produced from pasteurized milk were linked to a widespread outbreak of listeriosis in Germany. Furthermore, *Escherichia coli* is one of the most significant foodborne pathogens, causing disease in both humans and animals. Honish et al. (2005) reported that *E. coli* led to a cheese‐associated outbreak in Canada, resulting in two cases of Hemolytic Uremic Syndrome (HUS). These pathogens can also survive in soft cheese stored at 4°C (Ehsani et al. 2016). Moreover, the growth of foodborne molds, including *Penicillium* and *Aspergillus*, on cheese during ripening, curing, and refrigerated storage is a common problem for cheese manufacturers. Essential oils (EOs) are considered safe (GRAS) under the United States Food and Drug Administration (US FDA) regulations. They are recognized as natural green preservatives that can prevent the growth of foodborne pathogens (Hematizad et al. 2021). *Zataria multiflora* Boiss., known locally in Iran as “Avishan Shirazi” is a spice plant belonging to the Lamiaceae family. Its EO is used to extend the shelf life of foods and as a flavoring agent in various dishes. Additionally, its leaves are used in traditional medicine. Various studies have concluded that ZEO exhibits antimicrobial, antioxidant, antiseptic, antispasmodic, and antinociceptive effects (Fazeli et al. 2007; Gandomi et al. 2009; Hematizad et al. 2021; Misaghi and Basti 2007; Ruiz‐Navajas et al. 2013; Sharififar et al. 2007, 2010). Additionally, due to various factors such as the low water solubility of EOs, the dependence of their antimicrobial properties on concentration, and the potential to create undesirable sensory properties in foodstuffs, it is not recommended to add EO singly to foodstuffs (Hematizad et al. 2021). Therefore, using EO in combination with another technique to ensure food safety against pathogenic bacteria while maintaining optimal organoleptic properties could be a practical method. Recently, significant attempts have been made to extend nonthermal plasma‐based sterilization processes. This novel emerging method has attracted significant attention from scientists in the food industry because it does not alter the physical properties or original composition of foodstuffs (Lacombe et al. 2015; Mendes‐Oliveira et al. 2019).

During the last decade, several types of nonthermal (cold) atmospheric pressure plasmas, such as gliding arc discharge (GAD), corona discharges, one atmospheric uniform glow discharge, microhollow cathode discharges, plasma spray, dielectric barrier discharge (DBD), atmospheric pressure plasma jet (APPJ), and plasma needle, have been suggested for bio‐decontaminating foodstuffs, agricultural products, and biological material contact surfaces. These methods target different bacteria and fungi (yeasts and molds) and are used for quality control in agricultural products during storage (Sruthi et al. 2022; Kaavya et al. 2023; Shanker et al. 2023; Dasan et al. 2016; Nehra et al. 2008; Pankaj et al. 2013; Pawłat 2013; Ramazzina et al. 2015; Sen and Mutlu 2013). Among the methods of plasma generation at ambient temperature and atmospheric pressure, GAD holds the biggest advantage over other techniques due to its ease of use, low equipment costs, and affordability. In this reactor, the air flows between two metal electrodes, creating a potential difference that converts the arc discharge into a bulk plasma flame. Niemira and Sites (2008) used GAD for the inactivation of outbreak strains of *S. Stanley* and *E. coli* on agar plates and the surfaces of Golden Delicious apples. Moreau et al. (2007) applied GAD plasma for the inactivation of three representative strains of *Erwinia*, which are among the most important microorganisms affecting potato plants. Song et al. (2009) used atmospheric pressure plasma with different input powers (75, 100, 125, and 150 W) and different exposure times (60, 90, and 120 s) to treat a three‐strain cocktail of *L. monocytogenes* on cheese slices and ham. The calculated *D* values for APP treatments at various power levels (75, 100, 125, and 150 W) were 71.43, 62.50, 19.65, and 17.27 s for cheese slices, and 476.19, 87.72, 70.92, and 63.69 s for sliced hams, respectively. Regardless of plasma exposure time, no viable cells were observed in sliced cheese treated with 125 and 150 W of APP treatment, after 1 week, at the detection limit of 10^1^ log CFU/g. In the study by Burlica et al. (2010) a gliding arc reactor with a water spray apparatus reduced the remaining number of *E. coli* colonies growing on an agar substrate by 4 log units after 2 min of treatment with either argon or air‐containing water spray. The application of GAD plasma as a promising new approach for inhibiting foodborne microorganisms on various substrates and foods has been investigated by several studies (Kaur et al. 2024; Zhao et al. 2024, 2025). However, the simultaneous use of EO and plasma has not been investigated. Therefore, in this study, the effect of ZEO, native to Iran, along with gliding arc plasma with air at different treatment times, was investigated on sliced Iranian white cheese artificially contaminated with *A. flavus* , *L. monocytogenes* , and *E. coli* . Additionally, the qualitative characteristics and organoleptic properties of the sliced cheese were assessed. So far, no study has been conducted to investigate the combined effect of these two antimicrobial agents together to determine whether they have a synergistic effect.

## 2 Materials and Methods

### 2.1 Preparation of the Plant Material

*Zataria multiflora* Boiss. was collected in Shiraz province of Iran and identified by the Institute of Medicinal Plants, Medical University of Tehran, Iran.

### 2.2 Extraction and Gas Chromatography/Mass Spectroscopy Analysis of EO

Air‐dried aerial parts of the plant (200 g) were subjected to steam distillation for 3 h using a Clevenger‐type apparatus. The obtained EO was sealed under nitrogen gas and stored in airtight glass vials covered with aluminum foil at 4°C. The EO was analyzed by GC (ThermoQuest 2000, ThermoQuest, Finnigan, UK). The chromatography system was equipped with a DB5 capillary column (30 × 0.25 mm ID × 0.25‐μm film thickness). Data were acquired under the following conditions: initial temperature 50°C, program rate 2.5°C, final temperature 265°C, and injector temperature 250°C. The carrier gas was helium with a split ratio of 120. The EO was also analyzed by GC/MS (ThermoQuest) using the same capillary column and analytical conditions as mentioned earlier. The MS was run in electron ionization mode with an ionization energy of 70 eV (Parsaeimehr et al. 2010).

## 3 Preparation of Microbial Cultures

### 3.1 Preparation of Conidial Suspension

*Aspergillus flavus* fungus ATCC 5041 was obtained from the Department of Microbiology, Faculty of Veterinary Medicine, University of Tehran. It was cultured on PDA (potato dextrose agar; Merck, Darmstadt, Germany) for a week at 25°C to promote spore development. To collect the spores, a mixture of 0.05% Tween 80 (Merck, Darmstadt, Germany) and sterile physiological serum was used. The culture was gently scratched with a sterile inoculating loop. The spore suspension was quantified using a hemocytometer slide and then diluted with a 0.05% Tween 80 solution to reach a final concentration of 10^6^ ppm (Gandomi et al. 2009).

### 3.2 Bacterial Strains

The strains used in this study were Lyophilized cultures of *E. coli O*_*111*_ and *L. monocytogenes* (ATCC 19111) prepared by the Department of Microbiology, Faculty of Veterinary Medicine, University of Tehran, Tehran, Iran. The lyophilized cultures of these strains were grown twice in tubes containing 10 mL of Brain–Heart Infusion (BHI) broth medium (Merck, KGaA, Darmstadt, Germany) at 37°C for 24 h. For inoculation dose preparation, the optical density (OD) (absorbance) of the final BHI broth cultures of *L. monocytogenes* and *E. coli* was adjusted to 0.1 at 600 nm wavelength, using a Spectronic spectrophotometer (Milton Roy Company, USA). A population of 8 log CFU/mL *L. monocytogenes* and *E. coli* was equal to 0.1 optical density at 600 nm. This dilution was achieved by diluting the resulting cultures of these pathogens with 9 mL of sterile 0.1% peptone water. Finally, 100 μL of each suspension was inoculated at 10 points on the cheese sample surface (Hematizad et al. 2021; Khanjari et al. 2013).

### 3.3 Preparation of Cheese Samples

Iranian white cheese (Pegah Milk Co. Ltd., Tehran, Iran) was purchased from a local market and cut into 4 mm thick slices before being treated with GAD plasma. Before the inoculation test, the cheeses were gamma sterilized with an irradiation dose of 35 kGy by using a cobalt‐60 irradiator at the Atomic Energy Organization of Tehran, Iran.

### 3.4 Treatment With GAD Plasma Source

In this study, an ARC type of Glide cold plasma device from the brand Nik Fanavaran Plasma, made in Iran, was used. Cold plasma was created using the DBD method by generating a potential difference and gas flow. In this research, a voltage of 26 kV and power of 300 W were used. The process variables included time (2 and 5 min) and a mixture of nitrogen (78%), oxygen (21%), and hydrogen (1%) gases (air) with a flow rate of 10 L/min. A high sinusoidal electric potential difference at a frequency of 50 Hz was used to treat the samples. The main structure of the device consists of two cylindrical electrodes connected to the AC power source. After the gas entered the capsule and the appropriate electric potential difference was applied, plasma, the fourth state of matter, was formed and ejected from the nozzle in the form of a jet. The length of the plasma jet is 27 mm, and the distance between the samples and the nozzle is 4 mm. On the other hand, the samples were treated at a temperature of 25°C and a humidity of 70%. Before performing the experiments, the air and the chamber where the samples were located were sterilized with an ultraviolet lamp for at least 30 min (Darvish et al. 2020). Table 1 shows the sample code and treatment conditions.

**TABLE 1:** Treatment codes and description.

Treatment code	Time of plasma (min)	ZEO (ppm)
T0	0	0
T1	0	100
T2	2	0
T3	2	100
T4	5	0
T5	5	100

## 4 Microbial Analysis

### 4.1 Radial Growth Measurement

The effect of EO and atmospheric cold plasma on radial mycelium growth in cheese was measured. EO, at a concentration of 100 ppm, was added to cheese samples in sterile plates. Then, 10 μL of *A. flavus* spore suspension was inoculated in the center of the cheese samples. Then, the samples were subjected to GAD plasma for 2 and 5 min and kept at 4°C for 60 days. The average of two perpendicular diameters of the fungal colony was calculated at five periods (0, 15, 30, 45, and 60 days). All experiments were performed with three repetitions (Gandomi et al. 2009). The following formula was used to calculate the percentage of mold growth inhibition:

$$\text{Inhibition of growth}\;\left(\%\right):\left(A_{2}–A_{1}/A_{2}\right)\times 100$$

where *A*_2_ is the diameter of colony in untreated group and *A*_1_ is the diameter of the colony in the treated group.

### 4.2 Bacterial Count

After the plasma process, each cheese sample (10 g) was mixed and homogenized with 90 mL of sterile peptone water. After diluting all the samples, microbial cultures for *E. coli* were grown on Trypticase Soy Agar (TSA), while *Listeria* cultures were grown on TSA supplemented with 0.6% yeast extract. After surface culturing each dilution (100 μL) on the appropriate culture medium, all plates were incubated at 37°C for 48 h. Colonies were counted in all samples on days 0, 15, 30, 45, and 60 in two repetitions. The number of bacteria was reported as log CFU/mL (Yong et al. 2015).

## 5 Chemical Analysis

### 5.1 pH Measurment

One gram of the treated groups was homogenized with 9 mL of distilled water for 30 s at 16,000 rpm, and the pH level was measured with a pH meter (Yong et al. 2015). Experiments were performed with two repetitions on days 0, 15, 30, 45, and 60.

### 5.2 TBARS Assessment

First, 200 mL of 2‐TBARS reagent was dissolved in 100 mL of 1‐butanol. Then, 5 mL of this solution was transferred along with 5 mL of thiobarbituric acid (TBA) reagent into a test tube with a lid. After vortexing, the solution was placed in a hot water bath at 95°C for 120 min and then cooled. Absorbance was measured at a wavelength of 530 nm using a spectrophotometer. The amount of absorption was measured in the presence of a blank reagent, and the concentration of malondialdehyde (mg/kg) in the sample was determined using the following formula. All experiments were performed with two replications on days 0, 15, 30, 45, and 60 (Karungi et al. 2004).

$$\text{TBARS}\;\left(\mathrm{mg}/\mathrm{kg}\right)=\frac{50\times \left(\mathrm{As}–\mathrm{Ab}\right)}{200}$$

### 5.3 Sensory Evaluation

Sensory evaluation was conducted by a group of seven experts in this field. The cheese was cut into 2 × 30 × 30 mm pieces, and the samples were evaluated using a nine‐point hedonic scale based on appearance characteristics such as color, taste, smell, texture, and acceptability. The scale was as follows: 9 = excellent, 8 = very good, 7 = good, 6 = fairly good, 5 = neither good nor bad, 4 = fairly bad, 3 = bad, 2 = very bad, and 1 = extremely unfavorable. A white plastic tray with a random three‐digit number was given to each panelist to prepare the samples, and water was available for rinsing the mouth during the session. The acceptability of the samples was analyzed using SPSS 25 statistical software (Raoofi et al. 2023).

### 5.4 Statistical Analysis

All experiments were performed in duplicate. SPSS Version 25 software was used for the statistical analysis of the obtained results. Quantitative data analysis was performed using one‐way ANOVA, and the results were presented as mean and standard deviation. Tukey's test with significance (*p* ≤ 0.05) was used to compare the means. Meanwhile, sensory characteristics were analyzed using the Kruskal–Wallis statistical test.

## 6 Results and Discussion

### 6.1 GC/MS Analysis of ZEO

Shirazi thyme is one of the oldest medicinal herbs used by humans. In this study, the yellow‐colored EO of Shirazi thyme was obtained using the steam distillation method and analyzed using gas chromatography–mass spectrometry (GC/MS). The extraction yield of ZEO based on dry weight was 1.66%. In similar studies, the extraction yield of thyme EO has been reported to range from 1.20% to 1.73% (Golkar et al. 2020). Table 2 shows the ZEO composition identified by GC–MS. Carvacrol is the most important compound in ZEO (33.85%), which is consistent with the results reported by Mohammadpour et al. (2016) and Aminzare et al. (2017) who reported the presence of thymol (37.59% and 17.86%), carvacrol (33.65% and 36.62%) and p‐cymene (7.72% and 11.35%) in ZEO. Thirty compounds were identified, representing 99.15% of the total oil. The main components were carvacrol (33.85%), thymol (23.18%), p‐cymene (11.13%), (e)‐beta‐cymene (4.47%), gamma‐terpinene (3.25%), and caryophyllene (2.9%). Mahmoudvand et al. (2017) identified 22 compounds in *Z. multiflora* EO. Thymol, carvacrol, and p‐cymene exhibited the highest amounts among the identified compounds. Karami‐Osboo et al. (2023) also reported similar results.

**TABLE 2:** ZEO composition identified by GC–MS.

No	Components	RT (min)	Composition (%)
1	α‐Phellandrene	14.32	0.13
2	(E)‐b‐Ocimene	14.58	4.47
3	Camphene	15.18	0.29
4	1‐Octen‐3‐ol	16	0.45
5	3‐Octanone	16.07	0.44
6	β‐Pinene	16.32	0.44
7	3‐Octanone	16.51	0.42
8	β‐Myrcene	17.09	1.12
9	a‐Terpinene	18	1.08
10	*ρ*‐Cymene	18.18	11.13
11	1,8‐Cineole	18.46	1.24
12	c‐Terpinene	19.57	3.25
13	Linalool	21.07	0.74
14	Borneol	23.59	0.21
15	4‐Terpinolene	24.31	1.29
16	a‐Terpineol	25.03	0.91
17	Thymol methyl ether	26.56	2.34
18	Thymol	28.59	23.18
19	Carvacrol	29.13	33.85
20	Thymol acetate	30.59	1.37
21	Durenol	31.25	2.18
22	p‐tert‐butyl catechol	32.58	0.33
23	(E)‐Caryophyllene	33.57	2.9
24	Aromadendrene	34.54	1.28
25	a‐Humulene	35.06	0.21
26	Ledol	36.4	0.57
27	Spatolenol	38.59	1.03
28	Caryophyllene oxide	39.02	1.57
29	Isobutyl‐O‐phthalate	46.22	0.4
30	Cineron	48.57	0.33
	Total	—	99.15

### 6.2 Microbial Analysis

In this study, the combined effect of atmospheric gliding arc cold plasma (2 and 5 min of irradiation) in the presence and absence of Shirazi thyme EO (100 ppm) on the growth inhibition rate of *A. flavus* , *E. coli* , and *L. monocytogenes* on Iranian white cheese was examined during 60 days of storage in the refrigerator.

### 6.3 Effect of Plasma Treatment and ZEO on *A. flavus* in Iranian White Cheese

Table 3 illustrates the rate of radial growth of *A. flavus* in Iranian white cheese samples over a 60‐day storage period. In the control group, the mold's radial growth increased from 9 mm on day 0 to 80 mm on day 60. On day 0, plasma irradiation for 2 and 5 min, either alone or combined with EO, did not have a significant impact (*p* > 0.05) on the radial growth of mold compared to the control samples. The inhibitory effect of plasma varied depending on the duration of plasma irradiation. Longer irradiation times led to a greater reduction in the radial growth rate of mold. There was a statistically significant difference (*p* < 0.05) between the groups treated with plasma for 2 and 5 min. Among the plasma‐treated samples, cheeses exposed to 2 min of plasma showed the highest rate of radial mold growth, which was not significantly different (*p* > 0.05) from samples containing 100 ppm EO alone during the storage period.

**TABLE 3:** Radial growth (mm) of *A. flavus* in cheese during 60 days storage.

Sample	0	15	30	45	60
T0	9.0 ± 0.57^a^	44.0 ± 1.52^d^	69.3 ± 3.84^c^	80.0 ± 0.0d	80.0 ± 0.0^e^
T1	9.0 ± 0.57^a^	22.3 ± 0.88^bc^	34.7 ± 1.45b	51.7 ± 2.3c	69.0 ± 1.15^d^
T2	9.0 ± 0.57^a^	24.66 ± 0.88^c^	34.0 ± 1.73b	53.0 ± 2.3c	68.7 ± 1.20^d^
T3	9.0 ± 0.57^a^	17.3 ± 0.88^ab^	25.7 ± 0.88ab	40.0 ± 0.57b	53.7 ± 1.76^c^
T4	9.0 ± 0.57^a^	15.0 ± 2.08^a^	22.7 ± 2.72a	29.0 ± 1.15a	40.7 ± 1.45^b^
T5	9.0 ± 0.57^a^	12.0 ± 1.15^a^	16.3 ± 0.88a	23.3 ± 0.88a	28.7 ± 1.20^a^

*Note:* Different letters in each column indicate a significant statistical difference (*p* ≤ 0.05). Data are presented as the mean ± SD of triplicate determinations. T0 = Control, T1 = Sample treated with 100 ppm ZEO, T2 = Sample treated for 2 min radiation, T3 = Sample treated with 100 ppm ZEO and 2 min radiation, T4 = Sample treated for 5 min radiation, T5 = Sample treated with 100 ppm ZEO and 5 min radiation.

In a study consistent with the findings of the current investigation, Dasan et al. (2016) examined the impact of dry air plasma at atmospheric pressure on *A. flavus* and *A. parasiticus* inoculated on hazelnuts. The results indicated that after 5 min of plasma radiation (100 V and 25 kHz), there was a reduction in the logarithm of *A. flavus* and *A. parasiticus* with an inoculation dose of 10^6^ log CFU/g by 4.50 and 4.19, respectively. It was also noted that prolonging the duration of plasma radiation resulted in the formation of larger pores on the surface of mold spores, leading to the disruption of the spores' coating and eventual cell death. Within the air plasma, radicals derived from oxygen and nitrogen create an oxidative environment that denatures proteins in the spore coatings. As the integrity of the spore coatings is compromised, the core becomes susceptible to attack by radicals generated by the plasma. Another mechanism that contributes to the fatal damage of the spores is the accumulation of charged particles on their surface, coupled with the continuous bombardment of free radicals, resulting in the rupture of the cell wall. The efficacy of air plasma deactivation is significantly influenced by the power of the device and the duration of treatment. Generally, atmospheric air cold plasma affects both the surface and intracellular structures of *A. flavus* , leading to cell wall drying, polysaccharide oxidation, hyphae thinning, and the formation of wrinkles. Ultimately, lipid oxidation and sugar residue interactions on the surface disrupt the cell integrity, leading to cell death (Los et al. 2020).

Kim et al. (2014) conducted a study on the antimicrobial properties of cold plasma on red pepper powder contaminated with *A. flavus* . Their findings revealed a reduction of 2.5 log when atmospheric nitrogen plasma (900 W) was applied. Similar outcomes were observed in studies involving brown rice (Suhem et al. 2013), Kashar cheese (Akarca et al. 2023), and peanuts (Devi et al. 2017) treated with atmospheric cold plasma, resulting in a decrease in *A. flavus* count. It is important to note that air plasma serves as a source of active oxygen and nitrogen species. The process of plasma irradiation leads to the generation of free radicals, highly reactive chemicals, charged ions, and ultraviolet rays, which collectively possess the ability to deactivate microorganisms through various mechanisms (Machala et al. 2018). Esmaeili et al. (2023) conducted a study on the impact of cold plasma on reducing *A. flavus* in pistachios, finding the highest fungal reduction (5.14 log) after exposure to plasma. They observed that longer exposure times to plasma led to a more significant reduction in *A. flavus* , consistent with the findings of their research. In a separate investigation, Bagheri et al. (2020) assessed the efficacy of atmospheric cold plasma in deactivating *A. flavus* in military rations snacks, noting that this method has the potential to enhance microbiological safety by reducing total aflatoxin levels. Additionally, Oliveira et al. (2020) demonstrated that thyme EO completely inhibited the growth of *A. flavus* , suggesting its potential as an alternative to synthetic chemicals.

The samples treated with plasma for 5 min alone and in combination with EO exhibited the least amount of radial growth. A statistically significant variance (*p* < 0.05) was noted between the two groups treated with plasma for 2 min alone and in conjunction with EO on days 15, 45, and 60. The most substantial difference was observed after the maintenance period, reaching 15 mm. By the end of the storage period, a notable distinction (*p* < 0.05) was evident between the two groups treated with plasma for 5 min alone and in the presence of EO. Among the groups treated with plasma for 2 and 5 min in combination with EO, a statistically significant difference (*p* < 0.05) was observed after the storage period (45 and 60 days). Numerous studies have highlighted the antibacterial, antifungal, antiviral, and anti‐parasitic properties of ZEO, with its antifungal efficacy being well documented in several studies (Posgay et al. 2022). The antimicrobial impact of ZEO is attributed to the presence of phenolic compounds like carvacrol, thymol, and p‐cymene. The interaction between the active groups of phenolic compounds in ZEO and the cellular components of microorganisms results in the disruption of the cytoplasmic membrane, proton motive force, electron flow, active transport, coagulation of cell contents, and ultimately, cell death. Consequently, the combined use of ZEO and atmospheric cold plasma exhibited a synergistic effect in eradicating *A. flavus* (Pereira 2021). Previous studies have also reported the antifungal properties of thyme against *A. flavus* by other researchers (Moosavi‐Nasab et al. 2018; Oliveira et al. 2020; Pekmezovic et al. 2015).

### 6.4 Effect of Plasma Treatment and ZEO on *E. coli O*_*111*_ and *L. monocytogenes* in Iranian White Cheese

**FIGURE 1:**
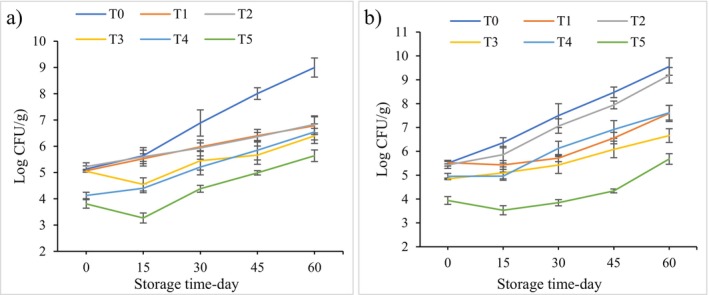
Change in the growth rate of *Escherichia coli* (a) and *Listeria monocytogenes* (b) bacteria on sliced Iranian white cheese during storage (mean ± SD). T0 = Control, T1 = Sample treated with 100 ppm ZEO, T2 = Sample treated for 2 min radiation, T3 = Sample treated with 100 ppm ZEO and 2 min radiation, T4 = Sample treated for 5 min radiation, T5 = Sample treated with 100 ppm ZEO and 5 min radiation.

The growth rate alteration of *E. coli O*_*111*_ and *L. monocytogenes* bacteria in Iranian white cheese samples treated with cold plasma and ZEO is illustrated in Figure 1a,b, respectively. The logarithm of the bacterial population in the control sample increased over time, as depicted in Figure 1. Statistically significant variances (*p* < 0.05) were observed. The most potent antimicrobial impact against bacteria was noted after a 5‐min exposure to EO radiation, resulting in a reduction of 3.35 and 3.87 log CFU/g in *E. coli* and *L. monocytogenes* , respectively, compared to the control groups at day 60. Plasma radiation exhibited a notable effect (*p* < 0.05) on the population of both bacteria, with a more pronounced decrease in the logarithm of the bacteria count observed as the radiation duration increased. These distinctions can be elucidated by the influence of Tween 80 nonionic emulsifier on the accessibility of EO and subsequently its antibacterial characteristics. These results demonstrate a satisfactory synergy between the antibacterial properties of atmospheric cold plasma and ZEO. In general, the antibacterial efficacy of plasma operates through three fundamental mechanisms: (1) Direct DNA disruption by ultraviolet photons (UV). (2) Cell membrane destruction by charged particles like (O^−^, OH^−^, H^+^, and e^−^) and (3) active species (e.g., O, O_3_, H_2_O_2_, and NO_2_) that cleave covalent bonds and instigate diverse chemical reactions (Schütz et al. 2021).

The findings of the current investigation indicate that cold atmospheric plasma exhibits greater efficacy in deactivating *E. coli* compared to *L. monocytogenes* . Various studies have demonstrated that the antimicrobial impact of plasma on Gram‐negative bacteria surpasses that on Gram‐positive bacteria (Lee et al. 2019), a phenomenon attributed to the peptidoglycan layer of Gram‐positive bacteria. Similar outcomes have been observed in the elimination of *E. coli* from milk (Gurol et al. 2012), ricotta cheese (Ricciardi et al. 2022), and cheese slices (Lee et al. 2012), as well as in the reduction of *E. coli* , *L. monocytogenes* , and *S. typhimurium* on cheddar cheese (Yong et al. 2015), *E. coli* , Salmonella, and *S. aureus* in Adobera cheese (Aguilar Uscanga et al. 2022), and *L. monocytogenes* in chicken muscle (Noriega et al. 2011). Ricciardi et al. (2022) reported that the population of *E. coli* in the control cheese became unacceptable in approximately 3 days, reaching 10^4^ log CFU/g very rapidly. Yong et al. (2015) reported that the populations of *E. coli* , *S. typhimurium* , and *L. monocytogenes* on the cheese slices were markedly diminished, with reductions measured to be 2.88, 3.11, and 2.26 log cfu/g, respectively, after 15 min of treatment.

The initial bacterial load, duration of plasma exposure, and food matrix are critical factors influencing antimicrobial efficacy. Nevertheless, our results indicate that this form of plasma can effectively reduce the microbial populations of *E. coli* and *L. monocytogenes* in Iranian white cheese, highlighting its potential utility in dairy products.

### 6.5 Effect of Plasma Treatment and ZEO on pH in Iranian White Cheese

**FIGURE 2:**
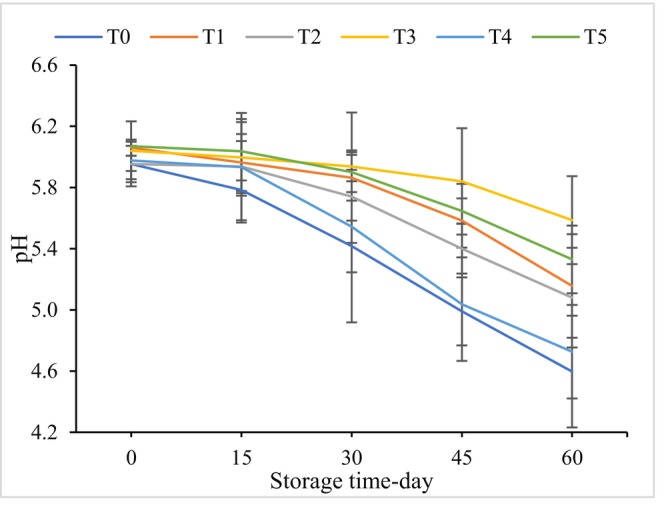
The pH value (mean ± SD) of sliced Iranian white cheese during storage. T0 = control, T1 = sample treated with 100 ppm ZEO, T2 = sample treated for 2 min radiation, T3 = sample treated with 100 ppm ZEO and 2 min radiation, T4 = sample treated for 5 min radiation, T5 = sample treated with 100 ppm ZEO and 5 min radiation.

pH is a critical parameter that significantly influences the quality of processed foods. Any significant alteration in the pH and acidity of cheese can have an undesirable impact on consumer acceptability (Ricciardi et al. 2022). The impact of gliding arc plasma radiation and ZEO on the pH value of Iranian white cheese during a 60‐day storage period is depicted in Figure 2. On day 0, there was no statistically significant difference (*p* > 0.05) observed in the pH of cheese samples. The changes in pH were examined alongside the fluctuations in microbial growth. Throughout the storage period, the pH values of all samples decreased. The control sample exhibited the highest reduction in pH, followed by the treated samples, with the sample treated with plasma for 5 min showing the most significant reduction. This pattern aligns with the findings of a study conducted by Yong et al. where slight changes in the pH values of cheddar cheese were observed up to 5 min of plasma radiation (Yong et al. 2015). In another study by Wan et al. the effect of atmospheric cold plasma with high voltage on the inactivation of *Listeria* inocula and *E. coli* in Queso Fresco cheese was evaluated. Their results indicated that plasma had a negligible effect on reducing the pH of cheese samples, which is consistent with the findings of the present study (Wan et al. 2021). Our results are in line with the majority of the literature, which reports that the formation of acid compounds from reactive nitrogen/oxygen species leads to a significant decrease in pH (Akarca et al. 2023; Korachi et al. 2010; Liao et al. 2020; Ricciardi et al. 2022; Shi et al. 2011; Yokoyama et al. 2019). For example, Akarca et al. reported a decrease in the pH of ricotta cheese from 6.69 to 6.34 by applying cold plasma (Akarca et al. 2023).

### 6.6 The Effect of Plasma Treatment and ZEO on TBARS in Iranian White Cheese

**FIGURE 3:**
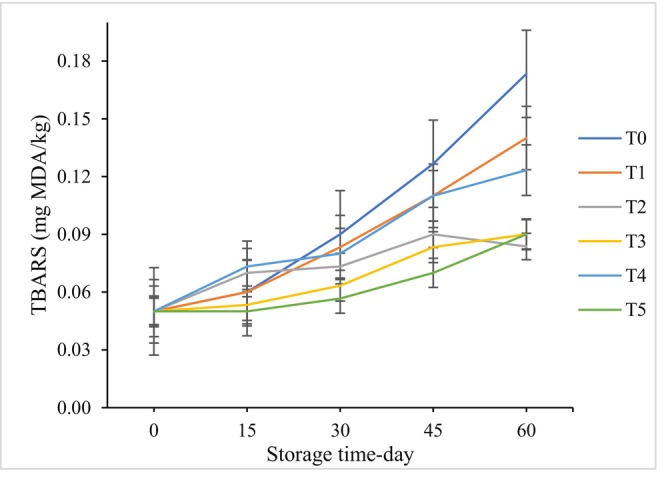
The TBARS value (mean ± SD) of sliced Iranian white cheese during storage. T0 = Control, T1 = Sample treated with 100 ppm ZEO, T2 = Sample treated for 2 min radiation, T3 = Sample treated with 100 ppm ZEO and 2 min radiation, T4 = Sample treated for 5 min radiation, T5 = Sample treated with 100 ppm ZEO and 5 min radiation.

Figure 3 illustrates the changes in the malondialdehyde content of Iranian white cheese during a 60‐day storage period. The results indicate that the level of malondialdehyde increased over time, with the control sample showing the most significant changes. Interestingly, the samples treated with 100 ppm ZEO and 5 min of plasma radiation exhibited the lowest increase in malondialdehyde content. This can be attributed to the formation of free radicals caused by plasma irradiation, which disrupts the function of fatty acids, particularly unsaturated fatty acids, leading to lipid oxidation. Additionally, the phenolic compounds in ZEO exhibited free radical scavenging activities which reduced the rate of oil oxidation (Mehdizadeh et al. 2018). This finding is consistent with a study by Jayasena et al. where the content of TBA in beef fillets increased after 10 min of plasma irradiation (Jayasena et al. 2015). Similarly, Yong et al. (2015) observed an increase in TBA content in cheddar cheese after 5 and 10 min of plasma radiation, supporting the results of the present study. However, no lipid oxidation was observed in bacon, cooked egg white, and yolk when exposed to atmospheric cold plasma (Kim et al. 2011; Lee et al. 2012). It is important to note that changes in TBA values are influenced by factors such as the type of plasma, carrier gas, and the lipid content and fatty acid composition of the treated sample, as highlighted (Aguilar Uscanga et al. 2022).

### 6.7 Effect of Plasma Treatment and ZEO on Sensory Characteristics in Iranian White Cheese

**FIGURE 4:**
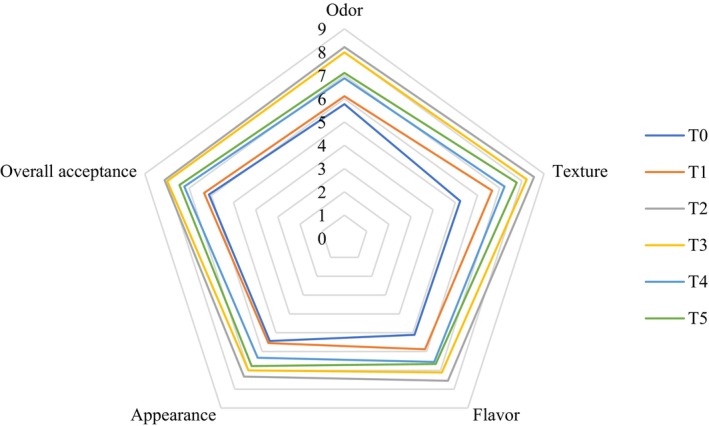
The sensory score of sliced Iranian white cheese during storage. T0 = Control, T1 = Sample treated with 100 ppm ZEO, T2 = Sample treated for 2 min radiation, T3 = Sample treated with 100 ppm ZEO and 2 min radiation, T4 = Sample treated for 5 min radiation, and T5 = Sample treated with 100 ppm ZEO and 5 min radiation.

In terms of the impact of gliding arc plasma radiation on sensory quality, data from the panel test of Iranian white cheese treated with ZEO on day 60 of storage is presented in Figure 4. The highest sensory characteristics score was observed in cheese samples radiated for 2 min. However, as the radiation duration increased to 5 min, the sensory properties score decreased. The control sample had the lowest sensory score, followed by the samples containing 100 ppm ZEO. On the other hand, radiation can induce changes in polypeptides. These findings are consistent with previous studies on sliced cheddar cheese (Yong et al. 2015), white cheese (Lee et al. 2012), and cheddar cheese (Ricciardi et al. 2022). Sensory characteristics such as taste, smell, and overall acceptance were identified as factors contributing to lipid oxidation in plasma‐treated cheeses, which can impact the sensory attributes of cheese. Numerous studies have demonstrated that the generation of free radicals, which are precursors of lipid hydroperoxides, leads to lipid oxidation and the formation of secondary oxidation products like alkanes, alkenes, aldehydes, alcohols, ketones, and acids (Yong et al. 2015). These secondary products of lipid oxidation result in off‐odors described as metallic, fishy, rancid, and oxidized. Sahebkar et al. (2020) explored the combined effect of plasma and plant EOs on extending the shelf life of chicken fillets. They found that the addition of plant EOs enhanced the sensory characteristics of chicken, while the aroma, taste, and overall acceptability decreased in samples treated with plasma. Their results indicated that the combined treatment reduced microbial growth in chicken fillets without negatively affecting smell, taste, and overall acceptance.

## 7 Conclusion

In this investigation, the impact of Gliding Arc plasma with varying durations (2 and 5 min) and ZEO at different concentrations (0 and 100 ppm) on the chemical parameters, microbial quality, and sensory attributes of Iranian white cheese during a 60‐day storage period was assessed. The findings revealed that an increase in radiation time resulted in a notable reduction in the radial growth rate of *A. flavus* , indicating a significant inhibitory effect (*p* < 0.05). The inhibitory effect of plasma on *E. coli* and *L. monocytogenes* exhibited a pattern that was dependent on the duration of plasma radiation. Throughout the storage period, there was a slight decrease in pH values. However, the level of malondialdehyde in Iranian white cheese increased over time. The highest sensory properties score was observed in cheese samples treated with 2 min of plasma radiation and 100 ppm ZEO. According to the results, it is suggested that the combined treatment of atmospheric cold plasma and ZEO has the potential to be utilized as a nonthermal technology and natural preservative for disinfecting cheese in the dairy industry. Importantly, this treatment does not adversely affect the sensory properties of the cheese, although optimization of conditions is necessary for industrial applications. One of the drawbacks of this study was the limited penetration ratio of the cold plasma in cheese.

## Author Contributions

**Mahdieh Raoofi Asl Soofiani:** investigation (equal), writing – original draft (equal). **Negin Noori:** project administration (equal), supervision (equal), writing – review and editing (equal). **Afshin Akhondzadeh Basti:** methodology (equal), supervision (equal), writing – review and editing (equal). **Hassan Gandomi:** data curation (equal), formal analysis (equal), methodology (equal), writing – review and editing (equal). **Hamed Ahari:** data curation (equal), formal analysis (equal), writing – review and editing (equal). **Mohammadreza Khani:** methodology (equal), writing – review and editing (equal).

## Conflicts of Interest

The authors declare no conflicts of interest.

## Data Availability

All data generated and analyzed during this study are available from the corresponding author upon reasonable request.
